# OFFSPRING EDUCATIONAL (DIS)ADVANTAGE, MECHANISMS, AND COGNITIVE FUNCTION IN KOREA

**DOI:** 10.1093/geroni/igad104.3503

**Published:** 2023-12-21

**Authors:** Hyungmin Cha

**Affiliations:** University of Southern California, Los Angeles, California, United States

## Abstract

Adult children’s education plays a significant role in parental cognitive well-being later in the life course. However, there is limited evidence in rapidly changing societies, leaving several key questions unanswered, including (1) whether the effects of offspring educational disadvantage can also flow upward, (2) whether these effects operate through similar explanatory mechanisms, such as financial, social, and informational support from their children, as seen in educational advantage, and (3) whether gender moderates these associations. Focusing specifically on Korea, a society where education has expanded rapidly and support from children is increasingly crucial, this study utilizes longitudinal data from the Korean Longitudinal Study of Aging (2006-2018) to demonstrate the role of offspring education in cognitive function during mid-to-late life. First, having adult children with a college degree or higher is associated with elevated levels of cognition and slower cognitive decline, whereas having adult children with less than a high school education is linked to lower levels of cognition and faster cognitive decline during mid-to-late life. Second, the association between adult children’s education and cognitive health, regardless of whether they have a college degree or less than a high school education, remains robust even after accounting for various other mediators. Third, while the effect of adult children’s education is more pronounced for mothers compared to fathers, children’s gender does not moderate the association. Together, I offer a novel explanation for the association between offspring educational advantage and disadvantage, and their connection to parental cognition and its subsequent decline in rapidly changing society.

